# Supplementary material for: Study on the Difference of Superhydrophobic Characteristics of Different Wood Furniture Substrates
Source: Polymers (Basel). 2023 Mar 25;15(7):1644. doi: 10.3390/polym15071644 (PMC10096626; doi:10.3390/polym15071644)
Supplement: Supplementary file 1 [file polymers-15-01644-s001.zip › polymers-2263856-supplementary.pdf]

Table S1. Average water contact Angle and standard deviation of 5, 10 and 20 mechanical wear-resisting tests

	5 times		10 times		20 times	
serial number	Average water contact Angle	Standard deviation	Average water contact Angle	Standard deviation	Average water contact Angle	Standard deviation
MW-A	140.3°	1.26	136.8°	1.14	127.5°	1.31
MW-B	141.9°	2.14	138.4°	1.94	130.5°	1.98
MW-C	140.5°	2.06	131.5°	2.14	123.8°	2.02
MW-D	141.5°	1.58	136.8°	1.95	133.6°	1.85
MW-E	133.8°	2.48	129.5°	2.04	123.8°	2.53
MW-F	139.5°	2.96	134.8°	2.58	132.4°	2.84
MW-G	135.8°	1.95	130.6°	1.54	120.8°	1.32
MW-H	142.0°	1.02	135.0°	1.24	120.7°	1.16

Table S2. Average water contact Angle and standard deviation of acid resistance test for 24h, 48h and 72h

	24h		48h		72h	
serial number	Average water contact Angle	Standard deviation	Average water contact Angle	Standard deviation	Average water contact Angle	Standard deviation
MW-A	140.5°	1.32	129.4°	1.28	132.8°	1.19
MW-B	144.0°	2.03	125.0°	2.24	113.6°	1.98
MW-C	135.3°	2.12	123.4°	1.89	126.2°	2.05
MW-D	131.8°	1.45	125.1°	1.68	115.6°	1.52
MW-E	123.4°	2.30	121.6°	2.02	120.5°	2.14
MW-F	129.2°	2.50	124.5°	2.14	121.7°	2.07
MW-G	141.5°	1.87	133.9°	1.45	128.8°	1.73
MW-H	142.6°	1.08	114.9°	1.12	119.5°	1.34

Table S3. Alkaline resistance test 24h, 48h, 72h average water contact Angle and standard deviation

	24h		48h		72h	
serial number	Average water contact Angle	Standard deviation	Average water contact Angle	Standard deviation	Average water contact Angle	Standard deviation
MW-A	147.6°	1.24	141.0°	1.24	138.0°	1.07
MW-B	142.3°	2.15	138.2°	1.89	131.2°	2.04
MW-C	141.8°	2.11	135.2°	2.34	131.6°	2.24
MW-D	140.5°	1.56	138.9°	1.89	133.9°	1.45
MW-E	143.6°	2.20	137.0°	2.04	130.0°	2.35
MW-F	138.9°	2.12	132.2°	2.35	126.4°	2.24
MW-G	145.8°	1.78	140.2°	1.58	134.3°	1.27
MW-H	141.0°	1.12	139.5°	1.24	131.2°	1.21
